# Supplementary material for: Filamentous recombinant human Tau activates primary astrocytes via an integrin receptor complex
Source: Nat Commun. 2021 Jan 4;12:95. doi: 10.1038/s41467-020-20322-w (PMC7782792; doi:10.1038/s41467-020-20322-w)
Supplement: Supplementary file 1 — Supplementary Information [file 41467_2020_20322_MOESM1_ESM.pdf]

Supplementary information

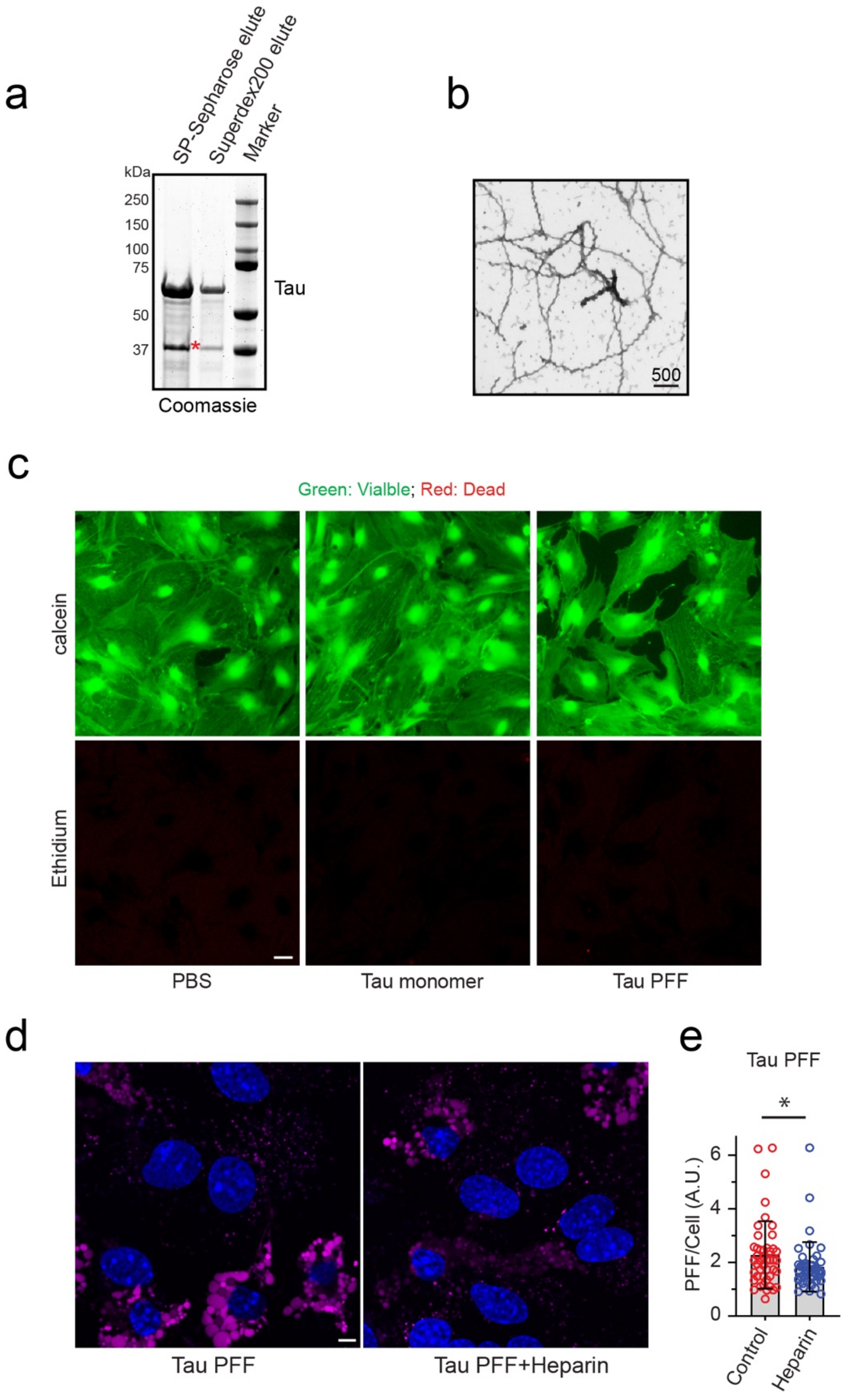

Figure S1. Tau PFF endocytosis by primary astrocytes is independent of HSPG.

(**a**) SDS-PAGE and Coomassie blue staining show the purified untagged Tau 2N4R protein. Protein eluates from SP-Sepharose and Superdex 200 were analyzed. Asterisk indicates a degradation product.

(**b**) A representative negative stain EM picture of the assembled Tau PFF before sonication. Scale bar, 500nm.

(**c**) Short-term treatment of astrocytes with Tau monomer or PFFs did not induce cell death. Primary astrocytes treated with PBS, Tau monomer or PFF (200 nM) for 6 h were stained with a green-fluorescent dye calcein-AM to label viable cells and a red-fluorescent dye ethidium homodimer-1 to label dead cells. Scale bar, 10 μm.

(**d**) Astrocytic uptake of Tau PFF is not significantly inhibited by heparin. Astrocytes were incubated with Alexa 594-conjugated Tau PFF (Magenta) for 2 h in the absence or presence of 50 μg/ml heparin. Cells were stained with Hoechst (Blue) before confocal imaging.

(**e**) Quantification of Tau PFF fluorescence signal in individual cells in experiments shown in **d**. Mean ±SEM, n=3 biologically independent experiments. *, $p<0.05$ by two-tailed unpaired student t-test.

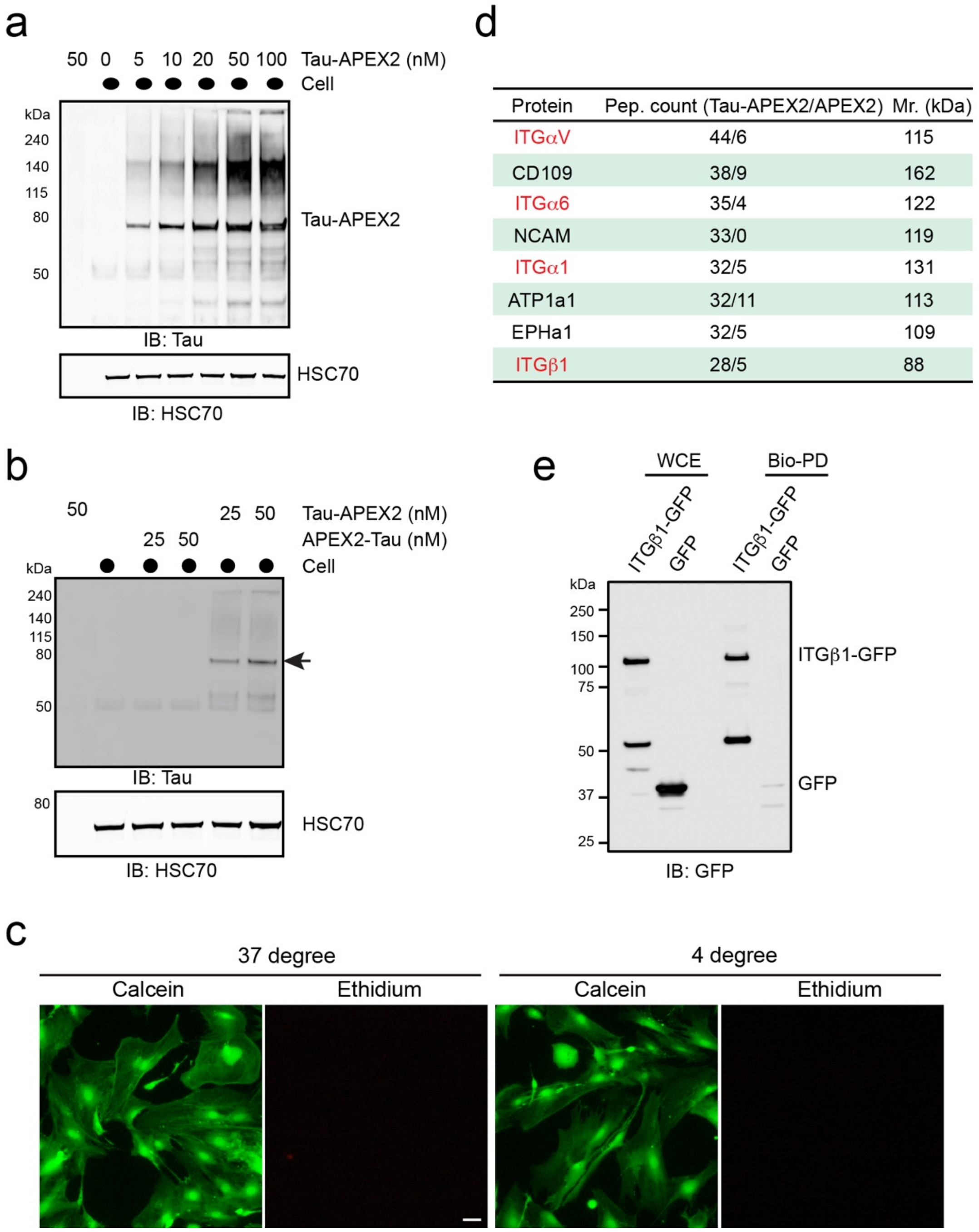

Protein	Pep. count (Tau-APEX2/APEX2)	Mr. (kDa)
ITGαV	44/6	115
CD109	38/9	162
ITGα6	35/4	122
NCAM	33/0	119
ITGα1	32/5	131
ATP1a1	32/11	113
EPHa1	32/5	109
ITGβ1	28/5	88

Figure S2. Identification of integrin αV/β1 as a Tau interactor in primary astrocytes.

(**a**) Tau-APEX2 binds to the cell surface in a dose dependent manner. Sh-SY5Y cells were incubated with the indicated amount of Tau-APEX2 on ice for 3 h. After washing, cells were lysed and cell lysates were analyzed by immunoblotting. As a negative control, 50 nM Tau PFF was incubated with buffer (no cells).

(**b**) Tau-APEX2 but not APEX2-Tau binds to the cell surface. Sh-SY5Y cells were incubated with the indicated amount of Tau protein on ice for 2 h, washed, and then lysed. Cell lysates were analyzed by immunoblotting. The arrow indicates Tau-APEX2.

(**c**) Short-term treatment of astrocytes with low temperature did not induce cell death. Primary astrocytes treated at 37 °C or 4 °C for 1 h were stained with calcein-AM and ethidium homodimer-1. Scale bar, 10 μm.

(**d**) A summary of the top candidates identified by mass spectrometry.

(**e**) Validation of Tau-APEX2-mediated biotinylation of ITGβ1. Tau-APEX2 was incubated with HEK293T cells transfected with either an empty vector (EV) or an ITGβ1-GFP-expressing plasmid followed by *in vitro* biotinylation. Whole cell extracts (WCE) were either directly analyzed by immunoblotting or first subjected to biotin pulldown (PD) before immunoblotting with GFP antibodies.

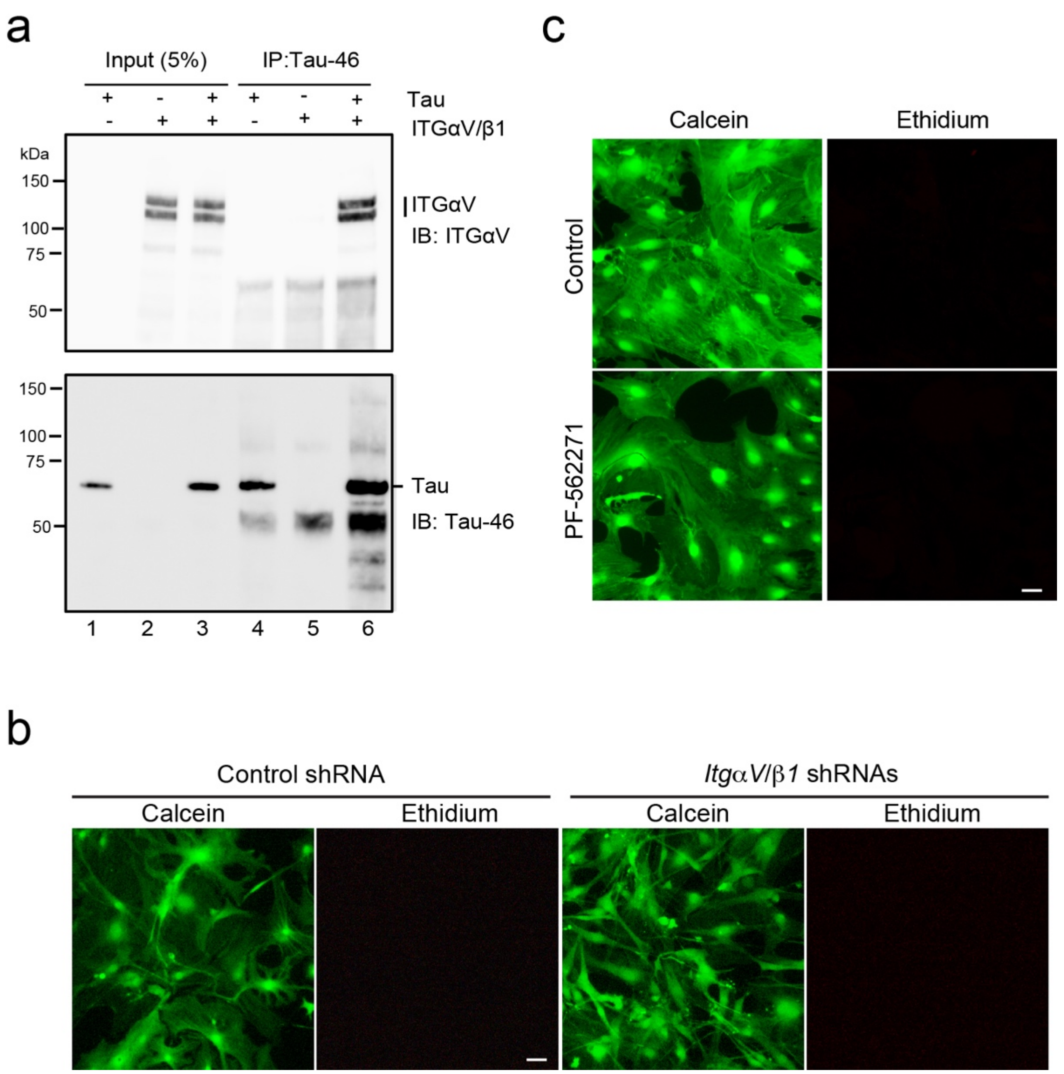

Figure S3. Tau binds integrin αV/β1 directly in vitro.

(**a**) Recombinant integrin αVβ1 (100 nM) was incubated with PBS as a control or 100 nM monomeric recombinant Tau protein as indicated. A fraction of the binding reaction (5% input) was analyzed directly by immunoblotting (lanes 1-3). The remaining samples were subjected to immunoprecipitation with Tau-46 antibodies before immunoblotting (lanes 4-6).

(**b**) The effect of integrin αV/β1 knockdown on astrocyte viability. Cells infected with lentiviruses expressing either control or integrin αV/β1 shRNAs for 72 h were stained with calcein-AM and ethidium homodimer-1. Scale bar, 10 μm.

(**c**) The effect of FAK inhibition on astrocyte viability. Cells treated with the indicated drugs for 8 h were stained with calcein-AM and ethidium homodimer-1. Scale bar, 10 μm.

a

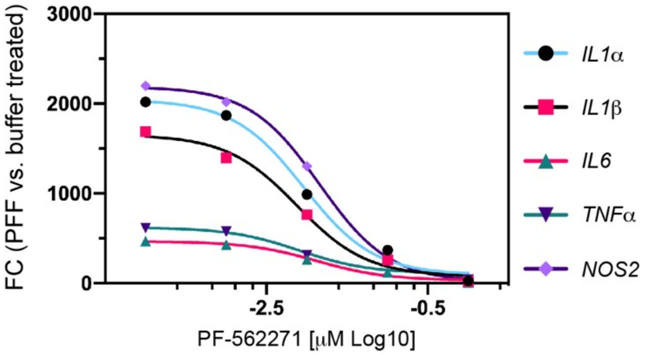

b

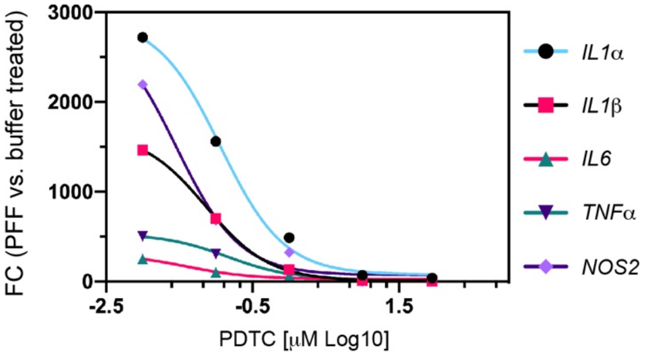

Figure S4. Dose dependent suppression of Tau-PFF-induced inflammation by PF-562271 and PDTC.

Immunopurified astrocytes were pre-treated with the indicated concentration of PF-562271 (**a**) or PDTC (**b**) for 1h and then treated with either PBS or Tau PFF. The expression of the indicated genes was determined by qRT-PCR and normalized to PBS-treated samples.

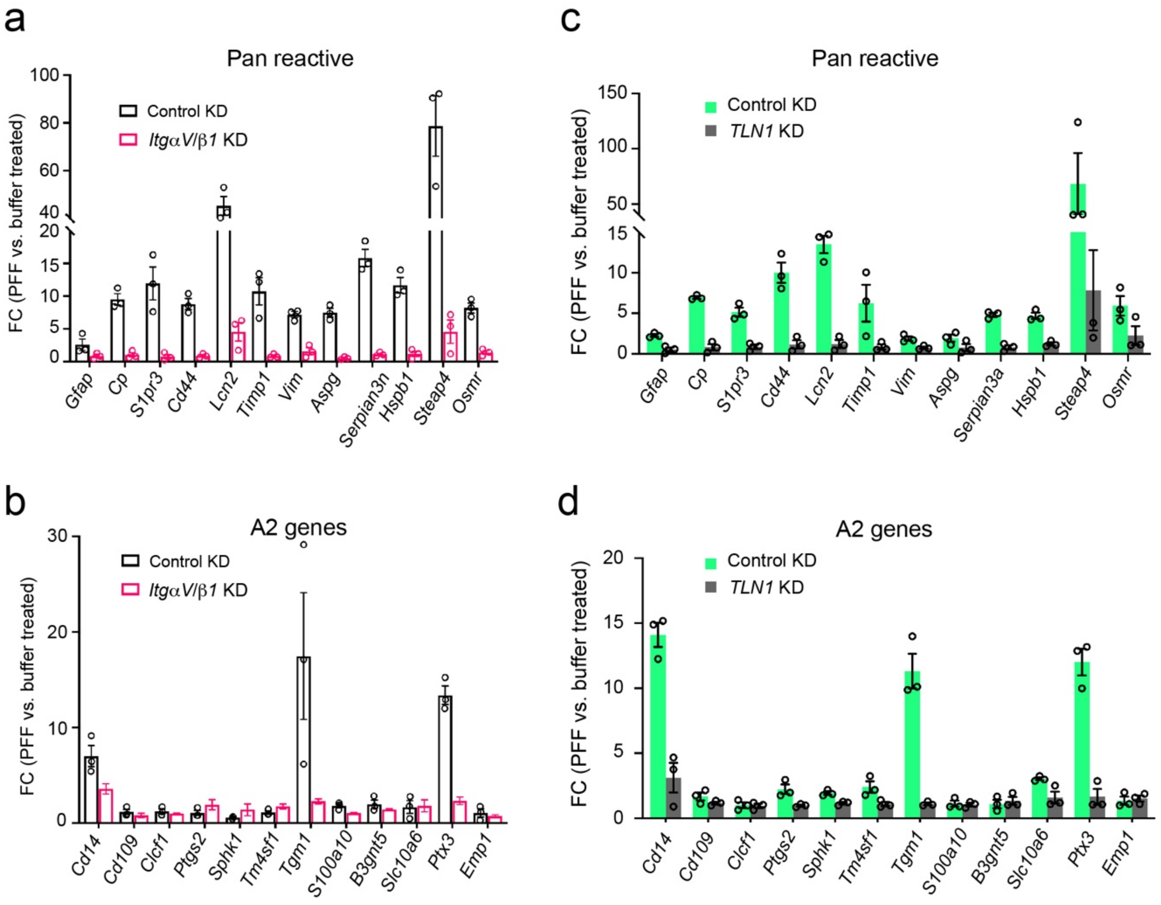

Figure S5. The effect of integrin αV/β1 and Talin1 knockdown on the expression of pan-reactive and A2 specific genes in astrocytes.

(**a, b**) Control or integrin αV/β1 knockdown astrocytes were treated with either PBS or Tau PFFs. The expression of the indicated genes was determined by qRT-PCR and normalized to PBS-treated samples. Mean ±SEM, n=3 independent experiments.

(**c**, **d**) Control or Talin1(TLN) knockdown immunopurified astrocytes were treated with either PBS or Tau PFFs. The expression of the indicated genes was determined by qRT-PCR and normalized to PBS-treated samples. Mean ±SEM, n=3 independent experiments.

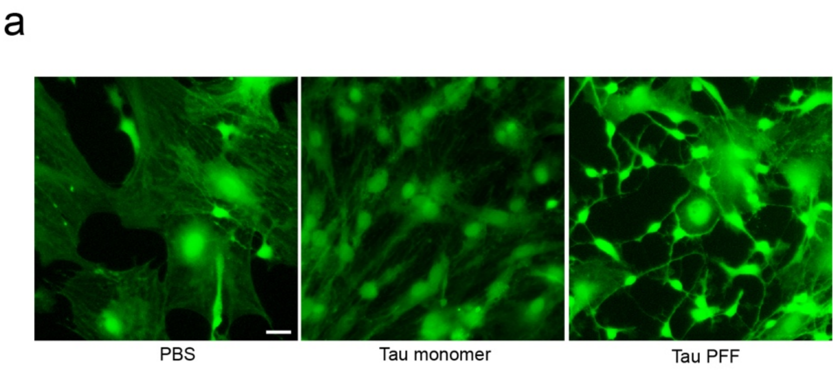

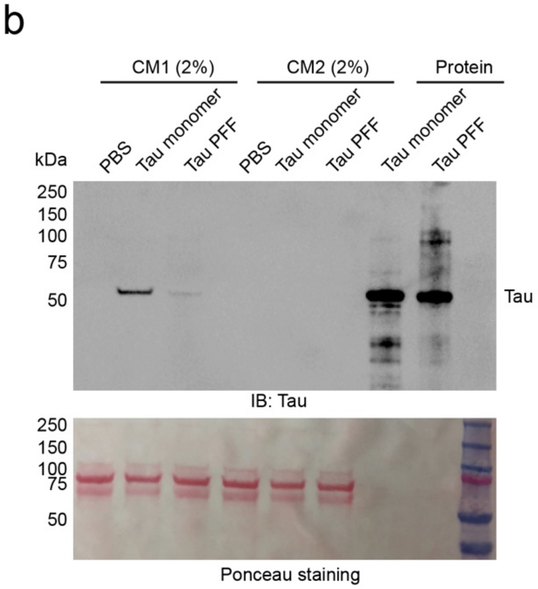

Figure S6 Tau PFF-treated astrocytes release a neurotoxic factor(s).

(**a**) Morphological changes in Tau PFF-treated astrocytes. Primary astrocytes were treated with PBS, Tau monomer or PFF (200nM, 6 h) and then replated in a new dish with fresh medium for 48 h before calcein-AM staining. Scale bar, 10 μm.

(**b**) No Tau carryover was detected in conditioned medium (CM) from Tau-treated astrocytes. Astrocytes were treated with PBS, monomeric Tau (200nM) or Tau PFF (200nM) for 6h. Conditioned medium (CM1) was harvested. Cells were then washed, trypsinized, and transferred to a new plate. After cell attachment, conditioned medium (CM2) was harvested. The CM1 and CM2 were analyzed by immunoblotting together with purified Tau monomer and PFF as a control.

SI Table 1 A list of reagents

REAGENTS	SOURCE
**PLASMIDS**	
pcDNA3 APEX2-NES	A gift from Alice Ting Addgene (# 49386) [1]
TAU/PET29B	A GIFT FROM PETER KLEIN ADDGENE (# 16316) [2]
pEF1-αV	A gift from Timothy Springer Addgene (# 27290) [3]
Integrin-β1-GFP	A gift from Martin Humphries Addgene (# 69804) [4]
pCMV-VSV-G	A gift from Bob Weinberg Addgene (#8454) [5]
pSPAX2	A gift from Didier Trono Addgene (#12260)
pEGFP-C1	Clonetech
ITGαV MISSION SHRNA SHRNA PLASMID DNA	Sigma SHCLND-NM_008402 TRCN0000066589
ITGβ1 MISSION SHRNA SHRNA PLASMID DNA	Sigma SHCLND-NM_010578 TRCN0000348624
TLN1 MISSION SHRNA SHRNA PLASMID DNA	Sigma SHCLND-NM_011602 TRCN0000108756
**CHEMICALS**	
PUROMYCIN	Sigma
PDTC	R&D
PH RODO-RED SUCCINIMIDYL ESTER	ThermoFisher Scientific
ALEXA 596 SUCCINIMIDYL ESTER	ThermoFisher Scientific
DYNASORE	TOCRIS
JASPLAKINOLIDE	TOCRIS
HOECHEST 33342	ThermoFisher Scientific
IPTG	TOCRIS
PF-562271	Selleckchem
BIOTIN-PHENOL	Iris-Biotech Cat # LS-3500.0250
**ANTIBODIES (Dilution)**	
INTEGRIN αV (1:1,000)	Abcam Cat # ab179475
INTEGRIN β1 (1:1,000)	Abcam Cat # ab52971
NFκB P65 (1:500)	Rockland Cat# 200-301-065
INTEGRIN β5 (20 μg/immune-panning)	R&D Systen Cat# AF3824

GFAP (1:250)	Proteintech Cat # 60190
TAU (TAU 46) (1:1,000)	Santa Cruz Cat # sc-32274
TAU (TAU H150) (2 μg/IP)	Santa Cruz Cat # sc-5587
BIOTIN (BTN,4) (1:1,000)	Thermo Fisher Cat # MA5-11251
FLAG (M2) (1:1,000)	Sigma Cat # F1804-200UG
GFP (B2) (1:500)	Santa Cruz Cat# SC-9996
HSP90 (1:1,000)	Santa Cruz Cat # sc-69703
CD45 (1.25 μg/immune-panning)	BD Biosciences Cat# 550539
ANTI-MOUSE IgG PEROXIDASE ANTIBODY (1:5,000)	Sigma Cat# A4416-1ML
ANTI-RABBIT IgG PEROXIDASE ANTIBODY (1:5,000)	Sigma Cat# A6154-1ML
GOAT ANTI-MOUSE IgG (H+L) ALEXA FLUOR$_{680}$ (1:10,000)	Thermo Fisher Cat# A21058
GOAT ANTI-RABBIT IgG (H&L) DYLIGHT$_{800}$ CONJUGATED (1:10,000)	Rockland Cat# 611-145-122
GOAT ANTI-MOUSE IgG ALEXA FLUOR$_{488}$ (1:1,000)	Thermo Fisher Cat# A21121
GOAT ANTI-RAT IgG (H + L) (80 μg/ immune-panning)	Jackson ImmunoResearch Cat# 112-005-167
DONKEY ANTI-SHEEP IGG (H + L) (80 μg/ immune-panning)	Jackson ImmunoResearch Cat# 713-005-147
**REGENTS**	
STREPTAVIDIN MAGNETIC BEADS	Pierce Cat # 88817
SODIUM ASCORBATE	VWR International Cat # 95035-692
TROLOX	Sigma-Aldrich Cat. # 238813-5G
SODIUM AZIDE	VWR International Cat # AA14314-22
HYDROGEN PEROXIDE 30% (WT/WT)	Sigma-Aldrich Cat # H1009-100ML
**PROTEIN**	
RECOMBINANT HUMAN INTEGRIN ALPHA 1 BETA 1 PROTEIN	R&D system

SI Table 2. qPCR primers used in the study

IL1α-F	5’-GGGAAGATTCTGAAGAAGAG
IL1α-R	5’-GAGTAACAGGATATTTAGAGTCG
IL1β-F	5’-TGTGAAATGCCACCTTTTGA
IL1β-R	5’-GTGCTCATGTCCTCATCCTG
IL6-F	5’-GACAACCACGGCCTTCCCTACTTC
IL6-R	5’-TCATTTCCACGATTTCCCAGAGA
TNFα-F	5’-CCGATGGGTTGTACCTTGTC
TNFα-R	5’-CGGACTCCGCAAAGTCTAAG
IL10-F	5’-AAGGCAGTGGAGCAGGTGAA
IL10-R	5’-CCAGCAGACTCAATACACAC
NOS2-F	5’-CACCTTGGAGTTCACCCAG
NOS2-R	5’-ACCACTCGTACTTGGGATGC
TGFβ1-F	5’-TACCATGCCAACTTCTGTCTGGGA
TGFβ1-R	5’-TGTGTTGGTTGTAGAGGGCAAGGA
CCL2-F	5’-TCAGCCAGATGCAGTTAACG
CCL2-R	5’-GATCCTCTTGTAGCTCTCCAGC
CCL3-F	5’-GACTGCCTGCTGCTTCT
CCL3-R	5’-GATCTGCCGGTTTCTCTTAG
CCL4-F	5’-CATGAAGCTCTGCGTGTCT
CCL4-R	5’-CTGCCGGGAGGTGTAA
CXCL10-F	5’-GCCGTCATTTTCTGCCTCAT
CXCL10-R	5’-GCTTCCCTATGGCCCTCATT
CCL12-F	5’-AGAATCACAAGCAGCCAGTGT

CCL12-R	5'-ATCCAAGTGGTTTATGGAATTCTTAAC
ITGβ1-F	5'-ATGCCAAATCTTGCGGAGAAT
ITGβ1-R	5'-TTTGCTGCGATTGGTGACATT
ITGαV-F	5'-CCTGAGACTGAAGAAGAC
ITGαV-R	5'-CCTTGCTGAATGAACTTG
ITGβ5-F	5'-TGACGAAGAACCACTATA
ITGβ5-R	5'-CTACTGTACGCATTGATAA
ITGα6-F	5'-AAGGAAGGATGTGGAGAC
ITGα6-R	5'-TTGAATTGGAAGGTAAGAGAAT
ITGα1-F	5'-AGCCTATCCTGAGACCTT
ITGα1-R	5'-TCTTATCTTCACCACAGTTCT
ITGα3-F	5'-GAGCTGTGGTTGGTGCTTG
ITGα3-R	5'-GCACTTCCACAAGAGGAGGAT
TLN1-F	5'-CTGGCCTCACAAGCCAAG
TLN1-R	5'-TTGATGTGAGCGCCTATCTCT
Iigp1-F	5'-GGGGCAATAGCTCATTGGTA
Iigp1-R	5'-ACCTCGAAGACATCCCCTTT
Gbp2-F	5'-GGGGTCACTGTCTGACCACT
Gbp2-R	5'-GGGAAACCTGGGATGAGATT
Fbln5-F	5'-CTTCAGATGCAAGCAACAA
Fbln5-R	5'-AGGCAGTGTCAGAGGCCTTA
Ugt1a-F	5'-CCTATGGGTCACTTGCCACT
Ugt1a-R	5'-AAAACCATGTTGGGCATGAT
Psmb8-F	5'-CAGTCCTGAAGAGGCCTACG
Psmb8-R	5'-CACTTTCACCCAACCGTCTT

Srgn-F	5’-GCAAGGTTATCCTGCTCGGA
Srgn-R	5’-TGGGAGGGCCGATGTTATTG
Amigo2-F	5’-GAGGCGACCATAATGTCGTT
Amigo2-R	5’-GCATCCAACAGTCCGATTCT
Clcf1-F	5’-CTTCAATCCTCCTCGACTGG
Clcf1-R	5’-TACGTCGGAGTTCAGCTGTG
Tgm1-F	5’-CTGTTGGTCCCGTCCCAAA
Tgm1-R	5’-GGACCTTCCATTGTGCCTGG
S100A10-F	5’-CCTCTGGCTGTGGACAAAAT
S100A10-R	5’-CTGCTCACAAGAAGCAGTGG
Sphk1-F	5’-GATGCATGAGGTGGTGAATG
Sphk1-R	5’-TGCTCGTACCCAGCATAGTG
Slc10a6-F	5’-GCTTCGGTGGTATGATGCTT
Slc10a6-R	5’-CCACAGGCTTTTCTGGTGAT
Tm4sf1-F	5’-GCCCAAGCATATTGTGGAGT
Tm4sf1-R	5’-AGGGTAGGATGTGGCACAAG
B3gnt5-F	5’-CGTGGGGCAATGAGAACTAT
B3gnt5-R	5’-CCCAGCTGAACTGAAGAAGG
VCAM-F	5’-TCTGGGAAGCTGGAACGAAG
VCAM-R	5’-CAAACACTTGACCGTGACCG
ITGαM-F	5’-ATGGACGCTGATGGCAATACC
ITGαM-R	5’-TCCCCATTCACGTCTCCCA
Serping1-F	5’-ACAGCCCCCTCTGAATTCTT
Serping1-R	5’-GGATGCTCTCCAAGTTGCTC
NF-κB-F	5’-CCAACGCCCTCTTCGACTAC

NF-κB-R	5’-GATCCCTCACGAGCTGAGC
GFAP-F	5’-AGAAAGGTTGAATCGCTGGA
GFAP-R	5’-CGGCGATAGTCGTTAGCTTC
CD109-F	5’-CACAGTCGGGAGCCCTAAAG
CD109-R	5’-GCAGCGATTTCGATGTCCAC
CD14-F	5’-GGACTGATCTCAGCCCTCTG
CD14-R	5’-GCTTCAGCCCAGTGAAAGAC
H2-D1-F	5’-TCCGAGATTGTAAAGCGTGAAGA
H2-D1-R	5’-ACAGGGCAGTGCAGGGATAG
H2-T23-F	5’-GGACCGCGAATGACATAGC
H2-T23-R	5’-GCACCTCAGGGTGACTTCAT
Emp1-F	5’-GAGACACTGGCCAGAAAAGC
Emp1-R	5’-TAAAAGGCAAGGGAATGCAC
Fkbp5-F	5’-TATGCTTATGGCTCGGCTGG
Fkbp5-R	5’-CAGCCTTCCAGGTGGACTTT
CD44-F	5’-TCAGGATAGCCCCACAACAAC
CD44-R	5’-GACTCCGTACCAGGCATCTTC
Serpina3n-F	5’-GTCTTTCAGGTGGTCCACAAGG
Serpina3n-R	5’-GCCAATCACAGCATAGAAGCG
CP-F	5’-GATGTTTCCCCAAACGCCTG
CP-R	5’-GTAGCTCTGAGACGATGCTTGA
S1pr3-F	5’-CTTGCAGAACGAGAGCCTGT
S1pr3-R	5’-CCTCAACAGTCCACGAGAGG
GFAP-F	5’-AACCGCATCACCATTCCTGT
GFAP-R	5’-TCCTTAATGACCTCGCCATCC

Lcn2-F	5'-CCGACACTGACTACGACCAG
Lcn2-R	5'-AATGCATTGGTCGGTGGGAA
Timp1-F	5'-CGCTAGAGCAGATACCACGA
Timp1-R	5'-CCAGGTCCGAGTTGCAGAAA
Vim-F	5'-GAGGAGATGAGGGAGTTGCG
Vim-R	5'-CTGCAATTTTTCTCGCAGCC
ASPG-F	5'-CAGGTGCCCAGGTTCCTATC
ASPG-R	5'-GTCCACCTTGGTTGTCCGAT
Hsbp1-F	5'-GAGATCACTGGCAAGCACGA
Hsbp1-R	5'-ATTGTGTGACTGCTTTGGGC
Steap4-F	5'-CAAACGCCGAGTACCTTGCT
Steap4-R	5'-CAGACAAACACCTGCCGACT
Osmr-F	5'-GTCATTCTGGACATGAAGAGGT
Osmr-R	5'-AATCACAGCGTTGGGTCTGA

References

1. Lam, S.S. *et al.* Directed evolution of APEX2 for electron microscopy and proximity labeling. *Nat Methods* **12**, 51-54 (2015).
2. Hedgepeth, C.M. *et al.* Activation of the Wnt signaling pathway: a molecular mechanism for lithium action. *Dev Biol* **185**, 82-91 (1997).
3. Takagi, J., Petre, B.M., Walz, T. & Springer, T.A. Global conformational rearrangements in integrin extracellular domains in outside-in and inside-out signaling. *Cell* **110**, 599-511 (2002).
4. Parsons, M., Messent, A.J., Humphries, J.D., Deakin, N.O. & Humphries, M.J. Quantification of integrin receptor agonism by fluorescence lifetime imaging. *J Cell Sci* **121**, 265-271 (2008).
5. Stewart, S.A. *et al.* Lentivirus-delivered stable gene silencing by RNAi in primary cells. *RNA* **9**, 493-501 (2003).
