# Supplementary material for: Filamentous recombinant human Tau activates primary astrocytes via an integrin receptor complex
Source: Nat Commun. 2021 Jan 4;12:95. doi: 10.1038/s41467-020-20322-w (PMC7782792; doi:10.1038/s41467-020-20322-w)
Supplement: Supplementary file 3 — Reporting Summary [file 41467_2020_20322_MOESM3_ESM.pdf]

nature research

Corresponding author(s): Yihong Ye

Last updated by author(s): Nov 25, 2020

Reporting Summary

Nature Research wishes to improve the reproducibility of the work that we publish. This form provides structure for consistency and transparency in reporting. For further information on Nature Research policies, see Authors & Referees and the Editorial Policy Checklist .

Statistics

For all statistical analyses, confirm that the following items are present in the figure legend, table legend, main text, or Methods section.

n/a	Confirmed	
☐	☒	The exact sample size (*n*) for each experimental group/condition, given as a discrete number and unit of measurement
☐	☒	A statement on whether measurements were taken from distinct samples or whether the same sample was measured repeatedly
☐	☒	The statistical test(s) used AND whether they are one- or two-sided<br>*Only common tests should be described solely by name; describe more complex techniques in the Methods section.*
☐	☒	A description of all covariates tested
☒	☐	A description of any assumptions or corrections, such as tests of normality and adjustment for multiple comparisons
☐	☒	A full description of the statistical parameters including central tendency (e.g. means) or other basic estimates (e.g. regression coefficient) AND variation (e.g. standard deviation) or associated estimates of uncertainty (e.g. confidence intervals)
☐	☒	For null hypothesis testing, the test statistic (e.g. *F*, *t*, *r*) with confidence intervals, effect sizes, degrees of freedom and *P* value noted<br>*Give P values as exact values whenever suitable.*
☒	☐	For Bayesian analysis, information on the choice of priors and Markov chain Monte Carlo settings
☒	☐	For hierarchical and complex designs, identification of the appropriate level for tests and full reporting of outcomes
☒	☐	Estimates of effect sizes (e.g. Cohen's *d*, Pearson's *r*), indicating how they were calculated

Our web collection on statistics for biologists contains articles on many of the points above.

Software and code

Policy information about availability of computer code

Data collection	Zeiss LSM780 with Zen, Bio-Rad CFX96 TM Real-Time System with CFX manager v3.1, LI-CORE scanner with Odyssey v3.0, Fuji LAS-4000 image reader
Data analysis	Image J, GraphPad Prism8, Excel, Adobe Photoshop, Fuji ImageGauge v3.0 software, LI-CORE Odyssey v3.0 software

For manuscripts utilizing custom algorithms or software that are central to the research but not yet described in published literature, software must be made available to editors/reviewers. We strongly encourage code deposition in a community repository (e.g. GitHub). See the Nature Research guidelines for submitting code & software for further information.

Data

Policy information about availability of data

All manuscripts must include a data availability statement. This statement should provide the following information, where applicable:

- Accession codes, unique identifiers, or web links for publicly available datasets
- A list of figures that have associated raw data
- A description of any restrictions on data availability

All data generated for this study are included in this article and its supplementary materials. Source data is available with this paper. All additional information is available from the corresponding author upon reasonable request.

Field-specific reporting

Please select the one below that is the best fit for your research. If you are not sure, read the appropriate sections before making your selection.

Life sciences study design

All studies must disclose on these points even when the disclosure is negative.

Sample size	Sample size was not pre-calculated because the goal of the study was to compare differences between different treated samples using statistical methods. The sample size was based on commonly adopted standard (>= 3 biological repeats). The number of independent repeats and statistical methods were reported in the figure legends.
Data exclusions	No data was excluded in this study.
Replication	All experiments (with the exception of the proximity-based ligation, which was done twice) were repeated at least 3 independent times. Error bars represent mean +/- SEM. All replication attempts were successful for the presented data.
Randomization	The goal of this study is to test the differential effect of different Tau variants on the same sample (primary astrocytes). Control and treated groups (drug or gene knockdown) were grown and treated in parallel in all the experiments reported in this manuscript. Thus, sample randomization is not applicable to this study. Image acquisition was performed with randomly selected cells/fields.
Blinding	Blinding was not used in this study because the experiments were all conducted and analyzed by one person. In qRT-PCR experiments, the differences between different sample groups were measured by a PCR machine, so no human bias was introduced. For imaging analyses, randomly chosen fields were imaged to avoid bias.

Reporting for specific materials, systems and methods

We require information from authors about some types of materials, experimental systems and methods used in many studies. Here, indicate whether each material, system or method listed is relevant to your study. If you are not sure if a list item applies to your research, read the appropriate section before selecting a response.

Materials & experimental systems

n/a	Involved in the study	
☐	☒	Antibodies
☐	☒	Eukaryotic cell lines
☒	☐	Palaeontology
☐	☒	Animals and other organisms
☒	☐	Human research participants
☒	☐	Clinical data

Methods

n/a	Involved in the study	
☒	☐	ChIP-seq
☒	☐	Flow cytometry
☒	☐	MRI-based neuroimaging

Antibodies

Antibodies used	Antibodies used in the study are listed in the Supplementary Table 1. Secondary antibodies are: ANTI-MOUSE IgG PEROXIDASE ANTIBODY (1:5,000), Sigma Cat# A4416-1ML; ANTI-RABBIT IgG PEROXIDASE ANTIBODY (1:5,000), Sigma Cat# A6154-1ML; GOAT ANTI-MOUSE IgG (H+L) ALEXA FLUOR680 (1:10,000), Thermo Fisher Cat# A21058; GOAT ANTI-RABBIT IgG (H&L) DYLIGHT800 CONJUGATED (1:10,000), Rockland Cat# 611-145-122; GOAT ANTI-MOUSE IgG ALEXA FLUOR488 (1:1,000), Thermo Fisher Cat# A21121; GOAT ANTI-RAT IgG (H + L) (80g/ immune-panning), Jackson ImmunoResearch Cat# 112-005-167; DONKEY ANTI-SHEEP IGG (H + L) (80g/ immune-panning), Jackson ImmunoResearch Cat# 713-005-147
Validation	Tau antibody was validated by immunoblotting using Tau recombinant protein as shown in Fig. 1f. Validation of other antibodies was done by Western blot by the manufactures.

Eukaryotic cell lines

Policy information about cell lines

Cell line source(s)	SH-SY5Y and HEK293T cells were purchased from ATCC. HEK293T cells were purchased from Thermo Fisher.
Authentication	Cell lines were authenticated at their source prior to acquisition, but not further authentication was performed.
Mycoplasma contamination	We routinely test mycoplasma contamination using a qPCR-based kit (Sigma). No mycoplasma contamination was found.
Commonly misidentified lines (See ICLAC register)	No commonly misidentified cell lines were used in the study.

Animals and other organisms

Policy information about studies involving animals; ARRIVE guidelines recommended for reporting animal research

Laboratory animals	New born (P0-P1) Wild-type C57BL/6J mice of mixed sex were used as the source for primary cells. Mice were housed in a temperature and humidity-controlled room (23-25 ℃, 40-60% humidity) with a 12 hr on-and-off light cycle.
Wild animals	No wildtype animals were used in the study.
Field-collected samples	None
Ethics oversight	Mice were merely used as a source for primary astrocytes and neurons. Study was conducted following the animal study protocol ASP K117-LMB-17 approved by the NIDDK Animal Care and Use Committee chaired by Dr. Constance Noguchi.

Note that full information on the approval of the study protocol must also be provided in the manuscript.
